# The relationship between synaptic density marker SV2A, glutamate and *N*-acetyl aspartate levels in healthy volunteers and schizophrenia: a multimodal PET and magnetic resonance spectroscopy brain imaging study

**DOI:** 10.1038/s41398-021-01515-3

**Published:** 2021-07-17

**Authors:** Ellis Chika Onwordi, Thomas Whitehurst, Ayla Mansur, Ben Statton, Alaine Berry, Marina Quinlan, Declan P. O’Regan, Maria Rogdaki, Tiago Reis Marques, Eugenii A. Rabiner, Roger N. Gunn, Anthony C. Vernon, Sridhar Natesan, Oliver D. Howes

**Affiliations:** 1 MRC London Institute of Medical Sciences, Imperial College London, Hammersmith Hospital Campus, London, W12 0NN UK; 2 Institute of Clinical Sciences (ICS), Faculty of Medicine, Imperial College London, London, W12 0NN UK; 3 Department of Psychosis Studies, Institute of Psychiatry, Psychology and Neuroscience, King’s College London, De Crespigny Park, London, SE5 8AF UK; 4 South London and Maudsley NHS Foundation Trust, Camberwell, London, SE5 8AF UK; 5 Department of Brain Sciences, Imperial College London, The Commonwealth Building, Hammersmith Hospital, Du Cane Road, London, W12 0NN UK; 6 Invicro, Burlington Danes Building, Du Cane Road, London, W12 0NN UK; 7 Centre for Neuroimaging Sciences, Institute of Psychiatry, Psychology and Neuroscience, King’s College London, De Crespigny Park, London, SE5 8AF UK; 8 Department of Basic and Clinical Neuroscience, Institute of Psychiatry, Psychology and Neuroscience, Maurice Wohl Clinical Neuroscience Institute, King’s College London, 5 Cutcombe Road, London, SE5 9RT UK; 9 MRC Centre for Neurodevelopmental Disorders, King’s College London, London, SE1 1UL UK

**Keywords:** Molecular neuroscience, Schizophrenia

## Abstract

Glutamatergic excitotoxicity is hypothesised to underlie synaptic loss in schizophrenia pathogenesis, but it is unknown whether synaptic markers are related to glutamatergic function in vivo. Additionally, it has been proposed that *N*-acetyl aspartate (NAA) levels reflect neuronal integrity. Here, we investigated whether synaptic vesicle glycoprotein 2 A (SV2A) levels are related to glutamatergic markers and NAA in healthy volunteers (HV) and schizophrenia patients (SCZ). Forty volunteers (SCZ *n* = 18, HV *n* = 22) underwent [^11^C]UCB-J positron emission tomography and proton magnetic resonance spectroscopy (^1^H-MRS) imaging in the left hippocampus and anterior cingulate cortex (ACC) to index [^11^C]UCB-J distribution volume ratio (DVR), and creatine-scaled glutamate (Glu/Cr), glutamate and glutamine (Glx/Cr) and NAA (NAA/Cr). In healthy volunteers, but not patients, [^11^C]UCB-J DVR was significantly positively correlated with Glu/Cr, in both the hippocampus and ACC. Furthermore, in healthy volunteers, but not patients, [^11^C]UCB-J DVR was significantly positively correlated with Glx/Cr, in both the hippocampus and ACC. There were no significant relationships between [^11^C]UCB-J DVR and NAA/Cr in the hippocampus or ACC in healthy volunteers or patients. Therefore, an appreciable proportion of the brain ^1^H-MRS glutamatergic signal is related to synaptic density in healthy volunteers. This relationship is not seen in schizophrenia, which, taken with lower synaptic marker levels, is consistent with lower levels of glutamatergic terminals and/or a lower proportion of glutamatergic relative to GABAergic terminals in the ACC in schizophrenia.

## Introduction

Converging lines of evidence suggest glutamatergic dysfunction is involved in schizophrenia pathophysiology [1–4]. Supporting this view, pharmacological modulation of glutamatergic function via *N*-methyl-D-aspartate receptor (NMDAR) antagonism can induce positive and negative symptoms and cognitive deficits in healthy subjects and exacerbate them in schizophrenia patients [5, 6], with large effect sizes [7]. Genomic studies have identified associations between schizophrenia and variants in genes encoding and/or regulating glutamatergic proteins, including NMDAR and activity-regulated cytoskeleton-associated protein complexes [8–11], which in turn regulate synaptic plasticity and cognition [12–14].

Glutamate levels can be measured in vivo using proton magnetic resonance spectroscopy (^1^H-MRS), which indexes total tissue glutamate, reflecting synaptic glutamate levels, the number of glutamatergic synapses, and extrasynaptic glutamate levels involved in neurometabolism [4, 15]. ^1^H-MRS studies have reported elevated glutamate and/or Glx (the sum of glutamate and its metabolite glutamine) levels in patients with schizophrenia in the anterior cingulate cortex (ACC) and medial temporal cortical regions including the hippocampus [16–20], although these data are not unequivocal and may be related to treatment response status.

It has been hypothesised that glutamatergic hyperactivity leads to excitotoxicity-mediated synaptic loss in schizophrenia [3, 21–23]. Supporting this, postmortem studies in schizophrenia have found that cortical glutamatergic neurons display reductions in synaptophysin (a marker of synaptic density), dendritic spine density and arborisation [24], and axospinous synaptic density [25] relative to controls. Furthermore, human-derived neurons from schizophrenia patients exhibit deficits in glutamatergic signalling, excitatory postsynaptic currents and synaptic connectivity relative to controls [26–28]. Whilst these findings indicate a link between glutamatergic function and synaptic markers in schizophrenia, it remains unknown if these are related in vivo in patients or in the healthy brain.

Synaptic density can now be measured in vivo using positron emission tomography (PET) with [^11^C]UCB-J, a radioligand specific for synaptic vesicle glycoprotein (SV2A), a protein selectively expressed in presynaptic terminals [29]. In a previous study from our laboratory, we found lower SV2A levels in patients relative to controls in the ACC and hippocampus [30], consistent with postmortem evidence for synaptic loss in these regions in schizophrenia [31–33]. If synaptic loss is secondary to ongoing glutamatergic excitotoxicity, SV2A and glutamate levels should be negatively associated. However, the relationship between SV2A and glutamate levels has not been investigated in vivo.

Therefore, we conducted a multimodal [^11^C]UCB-J PET and ^1^H-MRS imaging study to test the relationship between the proxy synaptic density marker SV2A and glutamate levels in vivo, hypothesising a negative relationship between the two in schizophrenia. Furthermore, levels of the neurometabolite *N*-acetyl aspartate (NAA), which may reflect neuronal integrity [34], are reduced in schizophrenia compared to controls in regions including the ACC and hippocampus [35], potentially secondary to aberrant synaptic elimination [36]. Therefore, we tested the hypothesis that SV2A and NAA levels would be directly associated in schizophrenia. We conducted these tests in the ACC and left hippocampus, given findings for synaptic, glutamatergic and NAA alterations in schizophrenia in these regions [16–19, 30–33, 35].

## Materials and methods

The study protocol was approved by the London-West London & GTAC Research Ethics Committee (reference: 16/LO/1941). Approval to administer radioactive material was obtained from the Administration of Radioactive Substances Advisory Committee. All volunteers provided written, informed consent before participating in the study, which was conducted in accordance with the Declaration of Helsinki.

### Schizophrenia patient and healthy volunteer recruitment

We recruited 18 patients from mental health services in London and 22 healthy volunteers through public advertisement. Inclusion criteria for all volunteers were: aged between 18 and 65 years old, demonstrated capacity to consent, and had a normal blood coagulation test in advance of arterial blood sampling. Age, gender, ethnicity and smoking status were recorded.

Patients were required to meet DSM-5 criteria for schizophrenia and to have had no changes in treatment for at least 4 weeks prior to the screening visit. Healthy volunteers were required to have no lifetime diagnosis of a mental disorder or family history of psychosis.

Exclusion criteria for all volunteers were: history of neurological disorder, head trauma resulting in a loss of consciousness, drug or alcohol dependence (except for nicotine dependence); significant medical disorder; taking a drug known to interact with SV2A (including levetiracetam, brivaracetam, loratadine or quinine) [37]; or contraindications to imaging.

### Clinical assessments

Schizophrenia patients underwent the Structured Clinical Interview for DSM-5 to confirm the diagnosis and screen for psychiatric co-morbidities. Healthy volunteers were screened with the Structured Clinical Interview for DSM-5 to exclude psychiatric illness, and screened to exclude family history of psychosis.

### MR imaging

All subjects underwent structural magnetic resonance imaging (MRI) to facilitate the anatomical delineation of regions of interest (ROIs) and to guide MRS voxel positioning. T1-weighted three-dimension magnetisation-prepared rapid acquisition gradient echo images were acquired on a Siemens Magnetom Prisma 3T scanner (Siemens, Erlangen, Germany) according to the following parameters: repetition time = 2300.0 ms, echo time = 2.28 ms, flip angle = 9 °, field of view = 256 × 256 mm, 176 sagittal slices of 1 mm thickness, voxel size = 1.0 × 1.0 × 1.0 mm.

### ^1^H-MRS acquisition

Single voxel ^1^H-MRS was acquired using the Point RESolved technique (TR = 3000 ms, TE = 30 ms, 96 averages, Vector size = 2048, Bandwidth = 2500 Hz). Prior to acquisition of the MRS the B0 homogeneity across the voxel was optimised automatically then fine-tuned manually using the first-order shim gradients to achieve a water linewidth of 18 Hz or less. A voxel measuring 20 × 20 × 20 mm was positioned in the ACC, in the midline immediately anterior to the genu of the corpus callosum (see Supplementary Fig. 1 for voxel placement and example spectrum). A second voxel measuring 20 × 20 × 15 mm was placed in the left hippocampus, angled parallel to the anterior horn of the temporal lobe and positioned just posterior to the amygdala, taking care to avoid the petrous bones (see Supplementary Fig. 2 for voxel placement and example spectrum).

### PET imaging

All participants underwent an [^11^C]UCB-J PET scan as described elsewhere [38]. Briefly, each subject had a CT scan for attenuation correction 2 min prior to radioligand injection. The study physician administered [¹¹C]UCB-J via an intravenous cannula as a smooth bolus injection over 20 s. PET data were acquired for 90 min using a Biograph 6 HiRez PET-CT scanner (Siemens, Erlangen, Germany).

### Arterial blood sampling

Radial arterial blood samples were collected throughout the PET scan to measure the arterial input function as described elsewhere [38]. Briefly, whole blood activity was measured using a continuous automatic blood sampling system (Allogg AB, Mariefred, Sweden). Discrete samples were taken at 10, 15, 20, 25, 30, 40, 50, 60, 70, 80 and 90 min after tracer injection. Total radioactivity concentration was evaluated in blood and plasma using a PerkinElmer 1470 10-well gamma counter. Discrete blood samples were used to determine the plasma radioactivity fraction constituted by unchanged parent radioligand using high-performance liquid chromatograph analysis. The [^11^C]UCB-J plasma free fraction was measured by ultrafiltration in triplicate using an arterial blood sample taken prior to tracer injection.

### Image analysis

We used distribution volume ratio (DVR) values as our primary [^11^C]UCB-J outcome measure for two reasons. First, DVR adjusts for non-specific tracer uptake using a reference region approach. Thus it is thought to reflect more closely the signal specific to SV2A in the region of interest (ROI) than volumes of distribution (*V*_T_) [39], which index both the radioligand concentration specifically bound to SV2A and the nondisplaceable uptake. Second, [^11^C]UCB-J DVR values show lower variability than *V*_T_ values [38] and so likely have greater sensitivity to detect group differences [40], as demonstrated in previous [^11^C]UCB-J analyses [30]. Notwithstanding this, we conducted exploratory analyses using the volumes of distribution values in addition to the primary analyses using DVR.

Processing and modelling were conducted using MIAKAT version 4.3.7 (http://www.miakat.org/MIAKAT2/index.html), implemented in MATLAB (Mathworks Inc., Natick, MA, USA) with functions from FSL (version 5.0.10; FMRIB, Oxford, UK) and Statistical and Parametric Mapping12 (SPM12, Wellcome Trust Centre for Neuroimaging, http://www.fil.ion.ucl.ac.uk/spm).

Each subject’s MRI underwent brain extraction using FSL, and grey matter segmentation and rigid-body coregistration to a standard reference space [41] using SPM12 as implemented via MIAKAT. The template brain image and associated Clinical Imaging Centre atlas [42] were then warped nonlinearly to the individual subject’s MRI where the ACC and left hippocampus were defined as ROIs. The centrum semiovale (CS) ROI was generated from the automated anatomical labelling template [43] according to parameters defined for its use as a reference region for nondisplaceable [^11^C]UCB-J binding [29].

PET images were registered to each subject’s MRI and motion-corrected using frame-to-frame rigid-body registration, with the 14th frame (acquired 9–11 min post-injection) as the reference frame. Regional time activity curves (TACs) were generated for each ROI.

Regional TAC and arterial input function data were analysed together using the one-tissue compartment model, which produces reliable estimates of [^11^C]UCB-J *V*_T_ [38, 44].

Grey matter masks were applied to the ROIs within MIAKAT to extract regional grey matter *V*_T_. Regional DVR was obtained by use of the CS as a pseudoreference region [29, 38].

MRS data were analysed using LC Model^®^ 6.3–1 L for automatic quantification of in vivo ^1^H-MR spectra [45] to index Glu, Glx and NAA concentrations. Glu, Glx and NAA values were scaled to creatine (Cr), which acts as an internal reference. Thus, we report Glu/Cr, Glx/Cr and NAA/Cr levels. Metabolite analyses were restricted to spectra with Cramér-Rao bounds ≤20% and signal-to-noise ratio ≥5%.

### Sample size and power calculation

We determined the minimum sample size required to test our primary hypothesis (that there is a negative relationship between synaptic density and glutamate measures). As no previous studies have examined the relationship between SV2A and glutamate measures in the living human brain, we determined that a strong relationship between SV2A and glutamate would be of clinical significance. The power calculation indicated a minimum sample size of 11 per group would have more than 80% power to detect a significant relationship at *r* of 0.7 between these variables, at *p* < 0.05 (two-tailed).

### Statistical analysis

Statistical analyses were performed using GraphPad Prism version 6.00 for Mac (GraphPad Software, La Jolla, California, USA (www.graphpad.com)) IBM SPSS Statistics, Version 25, and RStudio Version 1.1.456 (RStudio Team (2016), RStudio, Inc., Boston, MA (http://www.rstudio.com/)). We tested data for normality of distribution using the Shapiro–Wilk test. Our primary analysis tested the relationship between grey matter [^11^C]UCB-J DVR and Glu/Cr and NAA/Cr in the ACC and hippocampus in schizophrenia patients and healthy volunteers using Pearson product-moment correlation, with a Bonferroni-corrected alpha threshold for significance set at 0.0125 for the four tests (0.05/4) in each analysis to control the type I error rate. For completeness, we also conducted exploratory analyses testing the relationship between grey matter [^11^C]UCB-J DVR and Glx/Cr, and between grey matter [^11^C]UCB-J *V*_T_ and Glu/Cr, Glx/Cr and NAA/Cr, with an uncorrected alpha threshold for significance set at 0.05. Group differences in DVR, *V*_T_ and neurometabolite levels in the ACC and hippocampus, *V*_T_ in the CS, and clinico-demographic variables were assessed using two-tailed independent sample *t* tests for normally distributed data, Kolmogorov–Smirnov tests for nonparametric data and Chi-square test for categorical data. Grubbs test was used to identify potential outliers (alpha threshold = 0.05). When demographic variables differed significantly between groups, we tested whether these variables contributed significantly to DVR and neurometabolite levels. When this was the case, we fitted regression models to test whether significant relationships between [^11^C]UCB-J DVR and neurometabolites survived inclusion of these as covariates.

## Results

Forty volunteers (*n* = 18 with schizophrenia [SCZ, 15 male and 3 female] and 22 healthy volunteers [HV, 21 male and 1 female]) completed the study. The groups were well matched in terms of age, gender, ethnicity and recent cannabis use, and there were no significant group differences in mean radioactivity of the PET tracer administered, [^11^C]UCB-J plasma free fraction or CS [^11^C]UCB-J *V*_T_ (Table 1). A significantly greater proportion of the SCZ group were current smokers as compared to the healthy volunteer group (two-tailed Chi-square = 11.88, *p* = 0.0006). Seventeen patients were on antipsychotic medication, and none had co-morbid DSM-5 psychiatric diagnoses. PET data, but not MRS data, from 17 of the patients and 17 healthy volunteers have been been used in a separate analysis published recently [30]. The mean (SEM) interval between PET and MRS scans was 53.4 (9.6) days. Spectra quality data are reported in Supplementary Table 1.

**Table 1:** Clinico-demographic and imaging variables in healthy volunteer (HV) and schizophrenia (SCZ) groups. Values are mean (SEM) or number (*n*).

	HV	SCZ	*t*	Kolmogorov-Smirnov *D*	Chi-square	df	*p*
Age (years)	38.23 [2.59]	40.89 [2.75]	–	0.25	–	–	0.55
Male; Female (*n*)	21; 1	15; 3	–	–	1.62	–	0.20
Ethnicity							
White; black; other (n)	9; 9; 4	3; 13; 2	–	–	4.03	2	0.13
Current smoker (*n*)	3	12	–	–	11.88	1	0.0006
Cannabis use within last month (*n*)	0	2	–	–	2.57	1	0.11
Activity injected	264.40 [5.62]	243.60 [10.88]	–	0.32	–	–	0.25
Plasma free fraction	0.24 [0.005]	0.24 [0.005]	0.82	–	–	38.0	0.42
**CS**							
[^11^C]UCB-J *V*_T_	5.62 [0.12]	5.92 [0.34]	0.91	–	–	38.0	0.37
**ACC**							
[^11^C]UCB-J DVR	3.95 [0.10]	3.48 [0.18]	2.38	–	–	38.0	0.02
[^11^C]UCB-J *V*_T_	22.08 [0.67]	19.19 [0.76]	2.87	–	–	38.0	0.007
Glu/Cr	1.10 [0.02]	1.06 [0.02]	1.35	–	–	38.0	0.19
Glx/Cr	1.29 [0.03]	1.31 [0.05]	0.31	–	–	38.0	0.75
NAA/Cr	1.13 [0.02]	1.14 [0.02]	0.04	–	–	38.0	0.97
**Left hippocampus**							
[^11^C]UCB-J DVR	2.67 [0.08]	2.46 [0.14]	1.42	–	–	38.0	0.17
[^11^C]UCB-J *V*_T_	14.92 [0.50]	14.17 [0.54]	1.00	–	–	38.0	0.32
Glu/Cr	0.97 [0.03]	0.93 [0.04]	0.78	–	–	37.0	0.44
Glx/Cr	1.38 [0.06]	1.28 [0.07]	1.03	–	–	37.0	0.31
NAA/Cr	1.10 [0.03]	1.07 [0.04]	0.71	–	–	37.0	0.48

### [^11^C]UCB-J DVR and glutamate in the ACC

**Fig. 1:**
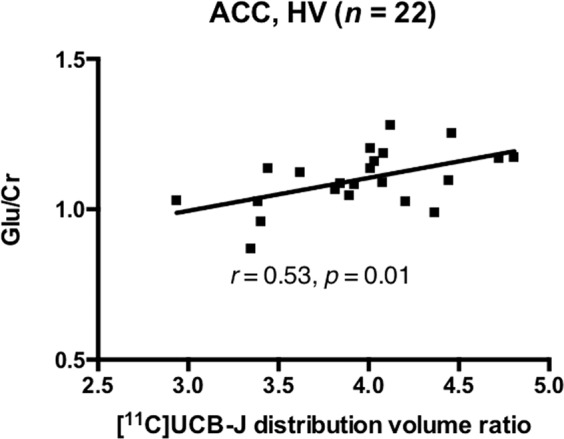
Synaptic density and glutamate in healthy volunteers. Significant positive relationship between [^11^C]UCB-J distribution volume ratio and Glu/Cr levels in the ACC in the healthy volunteer (HV) group. ACC = anterior cingulate cortex.

**Fig. 2:**
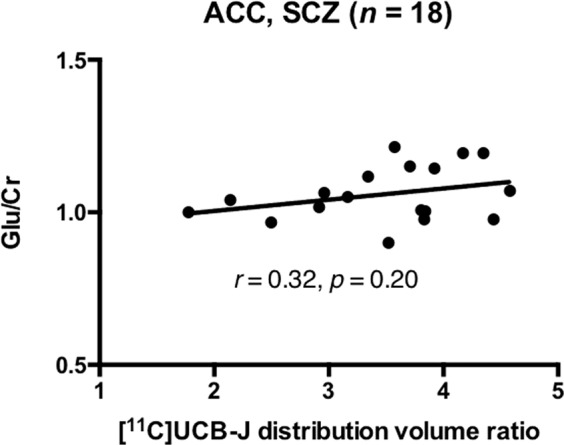
Synaptic density and glutamate in patients with schizophrenia. No significant relationship between [^11^C]UCB-J distribution volume ratio and Glu/Cr levels in the ACC in the schizophrenia (SCZ) group. ACC = anterior cingulate cortex.

Mean [^11^C]UCB-J DVR was significantly lower in the SCZ relative to the HV group in the ACC (*t* = 2.38, *p* = 0.02), with a large effect size (Cohen’s *d* = 0.8), but there were no significant differences between groups in Glu/Cr or Glx/Cr (Table 1). There was a significant positive relationship between [^11^C]UCB-J DVR and Glu/Cr in the HV (*n* = 22, *r* = 0.53, *p* = 0.01, Fig. 1), but not in the SCZ group (*n* = 18, *r* = 0.32, *p* = 0.20, Fig. 2). Our exploratory analysis found a significant positive relationship between [^11^C]UCB-J DVR and Glx/Cr in the ACC in the HV (*n* = 22, *r* = 0.72, *p* = 0.0002, Supplementary Fig. 3) but not in the SCZ group (*n* = 18, *r* = 0.05, *p* = 0.86, Supplementary Fig. 4).

Post-hoc analysis showed that there was no significant difference between patients and controls in the strength of the bivariate [^11^C]UCB-J DVR-Glu/Cr correlations (Fisher’s r-to-z: *z* = 0.74, *p* = 0.46), but there was a significant difference in the strength of the [^11^C]UCB-J DVR-Glx/Cr correlations (*z* = 2.48, *p* = 0.01).

### [^11^C]UCB-J DVR and NAA in the ACC

NAA/Cr levels were not significantly different between groups (Table 1). In the HV group, there was no significant relationship between [^11^C]UCB-J DVR and NAA/Cr in the ACC (*n* = 22, *r* = 0.07, *p* = 0.77, Supplementary Fig. 5). Although there was a positive relationship between [^11^C]UCB-J DVR and NAA/Cr in the ACC in the SCZ group (*n* = 18, *r* = 0.47, *p* = 0.048, Supplementary Fig. 6), this did not survive Bonferroni correction for multiple comparisons.

### [^11^C]UCB-J DVR and glutamate in the left hippocampus

Mean [^11^C]UCB-J DVR was not significantly altered in the SCZ relative to the HV group in the left hippocampus (*t* = 1.42, *p* = 0.17, Cohen’s *d* = 0.4). As Cramér-Rao bounds exceeded 20% for one schizophrenia patient’s hippocampal Glu/Cr and Glx/Cr data, these data were excluded from the analysis. There were no significant group differences in NAA/Cr, Glu/Cr or Glx/Cr in the left hippocampus (Table 1).

**Fig. 3:**
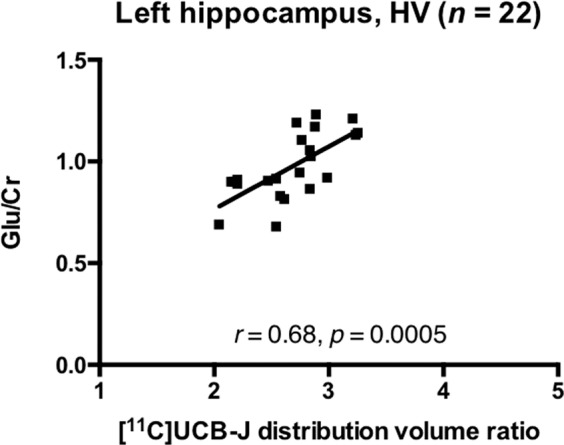
Synaptic density and glutamate in healthy volunteers. Significant positive relationship between [^11^C]UCB-J distribution volume ratio and Glu/Cr levels in the left hippocampus in the healthy volunteer (HV) group.

**Fig. 4:**
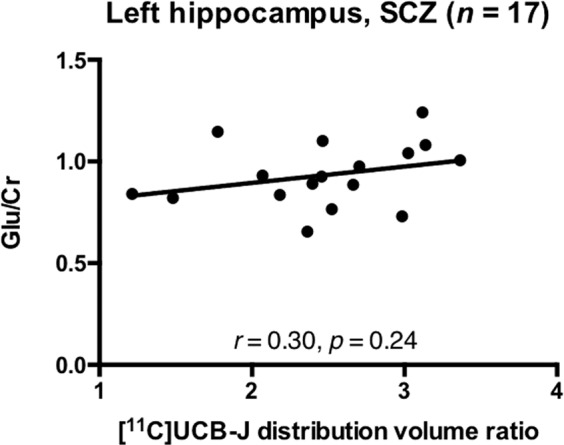
Synaptic density and glutamate in patients with schizophrenia. No significant relationship between [^11^C]UCB-J distribution volume ratio and Glu/Cr levels in the left hippocampus in the schizophrenia (SCZ) group.

There was a significant positive relationship between [^11^C]UCB-J DVR and Glu/Cr in the hippocampus in the HV group (*n* = 22, *r* = 0.68, *p* = 0.0005, Fig. 3), but not in the SCZ group (*n* = 17, *r* = 0.30, *p* = 0.24, Fig. 4). Our exploratory analysis found a significant relationship between [^11^C]UCB-J DVR and Glx/Cr in the hippocampus in the HV (*n* = 22, *r* = 0.48, *p* = 0.03, Supplementary Fig. 7) but not SCZ group (*n* = 17, *r* = 0.16, *p* = 0.53, Supplementary Fig. 8).

Post-hoc analyses showed that there were no significant differences between patients and controls in the strength of the bivariate [^11^C]UCB-J DVR-Glu/Cr (Fisher’s r-to-z: z = 1.46, *p* = 0.14) or [^11^C]UCB-J DVR-Glx/Cr (z = 1.00, *p* = 0.32) correlations.

### [^11^C]UCB-J DVR and NAA in the hippocampus

There were no significant relationships between [^11^C]UCB-J DVR and NAA/Cr in the hippocampus in the HV (*n* = 22, *r* = 0.23, *p* = 0.31, Supplementary Fig. 9) or SCZ (*n* = 18, *r* = −0.17, *p* = 0.50, Supplementary Fig. 10) groups.

### [^11^C]UCB-J V_T_ and neurometabolites

In the ACC, mean [^11^C]UCB-J *V*_T_ was significantly lower in the SCZ relative to the HV group (Table 1), with a large effect size (Cohen’s *d* = 0.9). There were no significant relationships between [^11^C]UCB-J *V*_T_ and Glu/Cr, Glx/Cr or NAA/Cr in the ACC in either group (Supplementary Material, Supplementary Figs. 11–16).

In the left hippocampus, mean [^11^C]UCB-J *V*_T_ was not significantly altered in the SCZ relative to the HV group (Cohen’s *d* = 0.3, Table 1). There was a significant positive relationship between [^11^C]UCB-J *V*_T_ and Glu/Cr in the hippocampus in the HV but not SCZ group (Supplementary Material, Supplementary Figs. 17–18), although exploratory, post-hoc analyses showed there was no significant difference in the strength of this relationship between groups (Fisher’s r-to-z: *z* = 1.06, *p* = 0.29). There were no significant relationships between [^11^C]UCB-J *V*_T_ and Glx/Cr or NAA/Cr (Supplementary Material, Supplementary Figs. 19–22) in either group in the hippocampus.

### Effect of smoking status on [^11^C]UCB-J DVR and neurometabolites

As smoking was more common in the SCZ than HV group, we conducted exploratory analyses to determine if smoking status could influence findings. There was no effect of smoking status on hippocampal Glu/Cr in the SCZ group, on hippocampal [^11^C]UCB-J DVR, Glx/Cr or NAA/Cr in either group, nor on ACC DVR, Glu/Cr, Glx/Cr or NAA/Cr in either group (see Supplementary Material). However, in the HV group, hippocampal Glu/Cr was significantly lower in smokers (smokers = 0.88 [0.03]; non-smokers = 0.99 [0.04]; *t* = 2.28, df = 13.2, *p* = 0.04). Nevertheless, the significant relationship between [^11^C]UCB-J DVR and Glu/Cr in the left hippocampus in the HV group remained after including smoking status in a regression model (*r* = 0.69, *p* = 0.002).

## Discussion

Our main finding is that [^11^C]UCB-J DVR is positively correlated with glutamate levels in the ACC and hippocampus in healthy volunteers, but not in patients with schizophrenia. [^11^C]UCB-J indexes SV2A, a presynaptic protein ubiquitously expressed in nerve terminals, and SV2A levels are closely related to other synaptic density markers such as synaptophysin [29, 46]. Thus, our findings extend ex vivo evidence that a large proportion of mammalian hippocampal and neocortical synapses are glutamatergic [47–53], by providing in vivo evidence for this in healthy humans as well. Moreover, our findings suggest synaptic density and glutamate levels are not related to the same degree in schizophrenia.

Our finding that [^11^C]UCB-J binding is significantly lower in the ACC in patients relative to controls is consistent with previous findings [30] in a cohort that largely overlaps with the sample in this study. In contrast, left hippocampal [^11^C]UCB-J *V*_T_ and DVR values were not significantly different between groups, although they were lower in schizophrenia in absolute terms. Our previous finding of significantly lower [^11^C]UCB-J DVR in the hippocampus in schizophrenia compared to healthy volunteers related to both hippocampi [30], whereas our findings here relate only to the left hippocampus. Moreover, compared to the ACC, hippocampal [^11^C]UCB-J *V*_T_ values show a lower signal-to-noise ratio with 42–90% greater variance in healthy hippocampal *V*_T_ values relative to total *V*_T_ [30, 54]. Thus, the lack of a significant group difference in left hippocampal [^11^C]UCB-J binding may reflect a lack of power. We did not find significant group differences in Glu/Cr, Glx/Cr and NAA/Cr, in contrast to findings from systematic reviews and meta-analyses [16, 18, 55, 56] but consistent with previous individual case-control studies of a similar size [57–61]. This likely reflects a lack of power to detect group differences of modest effect size as observed in meta-analysis. Importantly, it should be recognised that our study was not designed to detect group differences in these measures, but the relationship between [^11^C]UCB-J and the MRS measures.

### Strengths and limitations

We deployed multimodal imaging to investigate for the first time in vivo the relationship between a synaptic terminal marker and neurometabolites in the human brain. [^11^C]UCB-J shows good test–retest reproducibility, indicating it is a reliable imaging tool [44]. All but one of the schizophrenia subjects were taking antipsychotic medication. Recent studies found no effect of haloperidol or olanzapine administration on SV2A protein levels or specific binding in the rat brain, suggesting antipsychotic treatment is unlikely to affect the [^11^C]UCB-J measure in schizophrenia patients [30, 62]. There is some, albeit inconsistent evidence that antipsychotic drug administration may reduce glutamate levels in schizophrenia, with four of eight of published studies showing significant reductions (in the frontal and left temporal cortices, left thalamus and right striatum) following antipsychotic drug administration in schizophrenia while the other half show no significant change [63]. Notwithstanding this inconsistency, given that most of our patients were taking antipsychotics, it would be valuable to confirm our findings in unmedicated patients. There were significantly more smokers in the schizophrenia group than in the healthy volunteer group in our study. Furthermore, in the healthy volunteer group, we found that Glu/Cr levels were significantly lower in smokers. Previous studies have shown that smokers display lower cortical glutamate levels than non-smokers [64, 65]. Thus, our results may be confounded by differences in smoking levels across groups. However, we found that Glu/Cr levels did not differ significantly between groups and that smoking status did not alter the significant relationship between [^11^C]UCB-J DVR and Glu/Cr in the healthy volunteer group. Nonetheless, future studies should seek to control for smoking status between groups, and additionally to explore the relationship between quantity of smoking and glutamate levels. A potential limitation is that two subjects with schizophrenia had cannabis use within the last month, although this is a small and nonsignificant difference compared to healthy volunteers, and we excluded substance dependence in all volunteers through clinical assessment. Therefore, cannabis use is unlikely to have a marked effect on our findings. Nonetheless, it would be valuable to repeat these tests in a sample of patients entirely free of cannabis use. As our study does not probe GABA concentrations, we are unable to evaluate the relative densities of excitatory and inhibitory synapses in the healthy brain or in schizophrenia. In addition, at 3T, glutamate and glutamine are challenging to quantify due to considerable spectral overlap [66, 67]. Higher field strengths are needed to more precisely separate glutamate and glutamine signals. Creatine was used as an internal reference, which could affect analyses if there are creatine alterations in schizophrenia [68]. However, meta-analyses of brain creatine levels in schizophrenia have not found significant alterations, indicating this is unlikely to be a major factor in our analyses [55, 69]. As this was a cross-sectional study, it is not possible to delineate the precise mechanistic relationship between glutamate and SV2A in vivo. Animal models and patient-derived neuronal lines are needed to test the underlying mechanisms to establish directions of causality.

The primary outcome measure was DVR, estimated using the centrum semiovale as a reference region, given the evidence for very low specific binding levels in that region [29, 70]. The centrum semiovale is a white matter region largely devoid of SV2A and with very low [^11^C]UCB-J uptake [29, 38, 44, 70]. Nonetheless it displays a small amount of displacement of [^11^C]UCB-J uptake by levetiracetam, a drug selective for SV2A [29], suggesting some specific binding. Blocking studies have shown this is approximately 8% of that in grey matter, causing a slight underestimation of specific binding in grey matter, and white matter may not be an optimal reference region given its distinct tissue composition compared to grey matter [39]. Therefore, a group difference in centrum semiovale specific binding could bias our results. However, we did not find a significant difference between groups in centrum semiovale *V*_T_, consistent with our previous results [30]. This suggests differences in centrum semiovale specific binding are not driving our findings, and supports its suitability as a reference region in [^11^C]UCB-J PET in schizophrenia.

Although we found a significant difference between patients and controls in the strength of the [^11^C]UCB-J DVR-Glx/Cr correlations in the ACC, we found no such differences between groups in the hippocampus, nor between groups in the strength of the [^11^C]UCB-J DVR-Glu/Cr correlations in either region of interest, nor between groups in the strength of the [^11^C]UCB-J *V*_T_-Glu/Cr correlation in the hippocampus, using Fisher’s r-to-z transformation test. It should be noted that this study was not designed to test differences in the relative strengths of correlations, and is likely underpowered for these purposes, which would require a substantially greater difference in correlation strengths or a substantially larger study, or both.

### Implications for understanding the ^1^H-MRS glutamate signal

Our findings have implications for understanding the origin of the glutamate signal as captured by ^1^H-MRS. The highest brain glutamate concentrations are found in glutamatergic nerve terminals, with glial and extracellular glutamate levels kept low [71, 72]. Moreover, previous studies have reported that glutamate immunostaining intensity is significantly positively correlated with synaptic vesicle density (*r* = 0.94 in hippocampal mossy fibre terminals and *r* = 0.27 in Golgi cell terminals) [73] and that metabolic and neurotransmitter glutamate pools are tightly correlated [74]. These findings, taken with ours of a significant relationship between SV2A and glutamate in the ACC and hippocampus in the healthy brain, support the hypothesis that glutamate levels measured by ^1^H-MRS reflect the concentration of glutamate in the presynaptic neurotransmitter pool in the ACC and hippocampus [75–77]. It should also be noted that our findings are in keeping with preliminary in vivo evidence in a mixed sample of healthy volunteers and patients with Alzheimer’s disease showing a significant positive association between SV2A binding and metabotropic glutamate subtype 5 receptors (mGluR5) binding via radioligand [^18^F]FPEB, a potential marker of glutamatergic neurotransmission, and a significant mGluR5-by-region of interest interaction effect on [^11^C]UCB-J *V*_T_ [78]. Interestingly, in Mecca’s work, there was no effect of diagnosis on the relationship between mGluR5 and SV2A binding [78].

### Implications for understanding the ^1^H-MRS NAA signal

[^11^C]UCB-J binding was not related to NAA levels in either group in the ACC or hippocampus. NAA concentrations have been interpreted as potentially reflecting synaptic levels [36, 79–82]. However, our findings indicate NAA levels are not closely related to synaptic density in adulthood. NAA is synthesised in mitochondria and localised to synaptic terminals, dendritic arborisations, neuronal somata and fine axons [83]. Its levels may be affected by the broad range of processes in which it is involved, including myelin lipid synthesis, mitochondrial energy production, and synthesis of *N*-acetylaspartylglutamate [34]. Taken with the above, our findings that little of the variance in NAA levels measured in vivo is related to synaptic terminal levels in healthy volunteers or schizophrenia, indicate caution should be exercised before using NAA as proxy for synaptic terminal levels and/or synaptic elimination, and may suggest neurometabolism contributes more to its variance. Further work is warranted, however, to explore whether synaptic loss in the ACC is associated with lower levels of NAA measured by ^1^H-MRS in schizophrenia and other neuropsychiatric disorders.

### Implications for understanding schizophrenia

Our finding that [^11^C]UCB-J binding and glutamate levels are not related in either ROI in patients does not support our hypothesis that ongoing glutamatergic hyperactivity underlies synaptic alterations in schizophrenia. However, the lower synaptic density marker levels, taken with the loss of the normal relationship between this and glutamate levels in the ACC, could be explained by a loss of glutamatergic terminals in schizophrenia, such that a greater proportion of the remaining SV2A protein signal is from GABAergic synapses. This possibility is supported by postmortem [24, 25, 84] and other findings indicating excitatory-inhibitory imbalance in schizophrenia [85–88]. Another possibility is that a greater proportion of the glutamate signal is extra-synaptic, and thus unrelated to SV2A, in schizophrenia. Supporting this, there is evidence potentially for altered regulation of glutamate reuptake into synapses in schizophrenia [24, 71, 89]. These putative deficits are not mutually exclusive, and further work is needed to establish which is the main determinant of our findings. Notwithstanding this, our findings extend preclinical and clinical evidence for frontal cortical and hippocampal dysfunction in schizophrenia [90–95], to suggest this includes an altered relationship between synaptic terminal and glutamate levels.

## Conclusions

Levels of [^11^C]UCB-J binding, a synaptic terminal marker, are significantly positively correlated with Glu/Cr levels in the ACC and hippocampus in healthy volunteers, but not in schizophrenia. These findings indicate that an appreciable proportion of the ^1^H-MRS glutamatergic signal is related to synaptic density in healthy volunteers. They are not consistent with our hypothesis that current glutamatergic excitotoxicity leads to synaptic loss in schizophrenia, but do indicate the normal relationship between glutamate and synaptic terminal levels is disrupted, potentially due to lower levels of glutamatergic synapses and/or a reduced proportion of glutamatergic synapses in the anterior cingulate cortex in schizophrenia.

## Supplementary information

Synaptic density and glutamate in schizophrenia, Onwordi et al. 2021 - Supplementary Material

## Data Availability

Imaging and related clinical data will be made available upon reasonable request.

## Code availability

Code used to generate findings reported in this article will be made available upon reasonable request.
