# Supplementary material for: The relationship between synaptic density marker SV2A, glutamate and N-acetyl aspartate levels in healthy volunteers and schizophrenia: a multimodal PET and magnetic resonance spectroscopy brain imaging study
Source: Transl Psychiatry. 2021 Jul 17;11:393. doi: 10.1038/s41398-021-01515-3 (PMC8290006; doi:10.1038/s41398-021-01515-3)
Supplement: Supplementary file 1 — Synaptic density and glutamate in schizophrenia, Onwordi et al. 2021 - Supplementary Material [file 41398_2021_1515_MOESM1_ESM.doc]

**The relationship between synaptic density marker SV2A, glutamate and *N*-acetyl aspartate levels in healthy volunteers and schizophrenia: a multimodal PET and magnetic resonance spectroscopy brain imaging study**

**Onwordi et al.**

**Supplementary Material**

*Levene’s test for the equality of variances*

In the anterior cingulate cortex (ACC), there were significant differences between groups in the variances of [^11^C]UCB-J DVR (*F* = 4.67, *p* = 0.04) and Glx/Cr (*F* = 7.05, *p* = 0.01). There were no significant differences between groups in the variances of [^11^C]UCB-J *V*_T_ or other neurometabolites (*p* > 0.05). When using *t*-tests not assuming equal variances, [^11^C]UCB-J DVR remained significantly lower (*t* = 2.27, *p* = 0.03) and Glx remained similar (*t* = 0.30, *p* = 0.77) in the schizophrenia group compared to the healthy volunteer group in the ACC.

In the left hippocampus, there were no significant differences between groups in the variances of [^11^C]UCB-J DVR, *V*_T_ or neurometabolite levels (*p* > 0.05).

*[^11^C]UCB-J V_T_ and neurometabolites in the ACC*

There were no significant relationships between [^11^C]UCB-J *V*_T_ and Glu/Cr in the ACC in either group (HV, *n* = 22, *r* = 0.33, *p* = 0.14; SCZ, *n* = 18, *r* = 0.24, *p* = 0.34, Supplementary Figures 11 and 12).

There were no significant relationships between [^11^C]UCB-J *V*_T_ and Glx/Cr (HV, *n* = 22, *r* = 0.39, *p* = 0.07; SCZ, *n* = 18, *r* = 0.007, *p* = 0.98, Supplementary Figures 13 and 14), or between [^11^C]UCB-J *V*_T_ and NAA/Cr (HV, *n* = 22, *r* = -0.18, *p* = 0.43; SCZ, *n* = 18, *r* = -0.22, *p* = 0.37, Supplementary Figures 15 and 16) in either group in the ACC.

*[^11^C]UCB-J V_T_ and neurometabolites in the left hippocampus*

There was a significant positive relationship between [^11^C]UCB-J *V*_T_ and Glu/Cr in the hippocampus in the HV group (*n* = 22, *r* = 0.47, *p* = 0.03, Supplementary Figure 17), but not in the SCZ group (*n* = 17, *r* = 0.13, *p* = 0.61, Supplementary Figure 18).

There were no significant relationships between [^11^C]UCB-J *V*_T_ and Glx/Cr (HV, *n* = 22, *r* = 0.22, *p* = 0.32; SCZ, *n* = 17, *r* = -0.11, *p* = 0.68, Supplementary Figures 19 and 20), or between [^11^C]UCB-J *V*_T_ and NAA/Cr (HV, *n* = 22, *r* = -0.14, *p* = 0.53; SCZ *n* = 18, *r* = 0.02, *p* = 0.93, Supplementary Figures 21 and 22) in either group in the hippocampus.

*Effect of smoking status on [^11^C]UCB-J DVR and neurometabolites*

In the ACC, in the HV group, there was no significant difference between smokers and non-smokers in DVR (smokers = 3.71 [0.19]; non-smokers = 3.99 [0.11]; *t* = 0.95, df = 20, *p* = 0.35 [two-tailed independent samples t-test]), Glu/Cr (smokers = 1.12 [0.01]; non-smokers = 1.10 [0.02]; *t* = -0.32, df = 20, *p* = 0.75), Glx/Cr (smokers = 1.27 [0.01]; non-smokers = 1.29 [0.03]; *t* = 0.31, df = 20, *p* = 0.76), or NAA/Cr (smokers = 1.19 [0.04]; non-smokers = 1.13 [0.02]; *t* = -1.22, df = 20, *p* = 0.24).

In the ACC, in the SCZ group, there was no significant difference between smokers and non-smokers in DVR (smokers = 3.31 [0.23]; non-smokers = 3.81 [0.28]; *t* = 1.31, df = 16, *p* = 0.21 [two-tailed independent samples t-test]), Glu/Cr (smokers = 1.06 [0.03]; non-smokers = 1.05 [0.04]; *t* = -0.38, df = 16, *p* = 0.71), Glx/Cr (smokers = 1.30 [0.05]; non-smokers = 1.33 [0.11]; *t* = 0.26, df = 16, *p* = 0.80), or NAA/Cr (smokers = 1.15 [0.02]; non-smokers = 1.10 [0.05]; *t* = -0.99, df = 16, *p* = 0.34).

In the hippocampus, in the HV group, Glu/Cr was significantly lower in smokers (smokers = 0.88 [0.03]; non-smokers = 0.99 [0.04]; *t* = 2.28, df = 13.2, *p* = 0.04 [two-tailed independent samples t-test]). However, there was no significant difference between smokers and non-smokers in DVR (smokers = 2.44 [0.13]; non-smokers = 2.71 [0.08]; *t* = 1.25, df = 20, *p* = 0.23 [two-tailed independent samples t-test]), Glx/Cr (smokers = 1.27 [0.06]; non-smokers = 1.40 [0.07]; *t* = 0.65, df = 20, *p* = 0.52), or NAA/Cr (smokers = 1.05 [0.03]; non-smokers = 1.11 [0.03]; *t* = 0.82, df = 20, *p* = 0.42).

The significant relationship between [^11^C]UCB-J DVR and Glu/Cr in the left hippocampus in the HV group remained after including smoking status in a regression model *r* = 0.69, *p* = 0.002.

In the hippocampus, in the SCZ group, there was no significant difference between smokers and non-smokers in DVR (smokers = 2.38 [0.17]; non-smokers = 2.63 [0.21]; *t* = 0.87, df = 16, *p* = 0.40 [two-tailed independent samples t-test]), Glu/Cr (smokers = 0.91 [0.05]; non-smokers = 0.97 [0.06]; *t* = 0.68, df = 15, *p* = 0.50), Glx/Cr (smokers = 1.32 [0.08]; non-smokers = 1.21 [0.13]; *t* = -0.81, df = 15, *p* = 0.43), or NAA/Cr (smokers = 1.07 [0.05]; non-smokers = 1.06 [0.08]; *t* = -0.12, df = 16, *p* = 0.91).

Supplementary Table 1 – spectral quality measures.

|  | Healthy volunteers | Schizophrenia |
| --- | --- | --- |
| *Anterior cingulate cortex* |  | |
| FWHM | 0.04 (0.002) | 0.06 (0.005) |
| SNR | 45.68 (1.064) | 40.00 (1.561) |
| Glu CRLB | 4.68 (0.10) | 5.17 (0.19) |
| Glx CRLB | 5.77 (0.25) | 6.50 (0.29) |
| NAA CRLB | 2.05 (0.08) | 2.39 (0.18) |
| *Left hippocampus* |  | |
| FWHM | 0.06 (0.003) | 0.07 (0.003) |
| SNR | 14.59 (0.62) | 14.17 (0.80) |
| Glu CRLB | 10.55 (0.50) | 10.41 (0.49); n = 17 |
| Glx CRLB | 10.82 (0.43) | 11.71 (0.74); n = 17 |
| NAA CRLB | 4.50 (0.31) | 4.78 (0.76) |

Spectral line-width as full-width at half-maximum in parts per million (FWHM); signal-to-noise ratio (SNR); Cramér Rao Lower Bounds (CRLB); glutamate (Glu); glutamate plus glutamine (Glx); *N*-acetyl aspartate (NAA). Data are presented as mean (SEM).


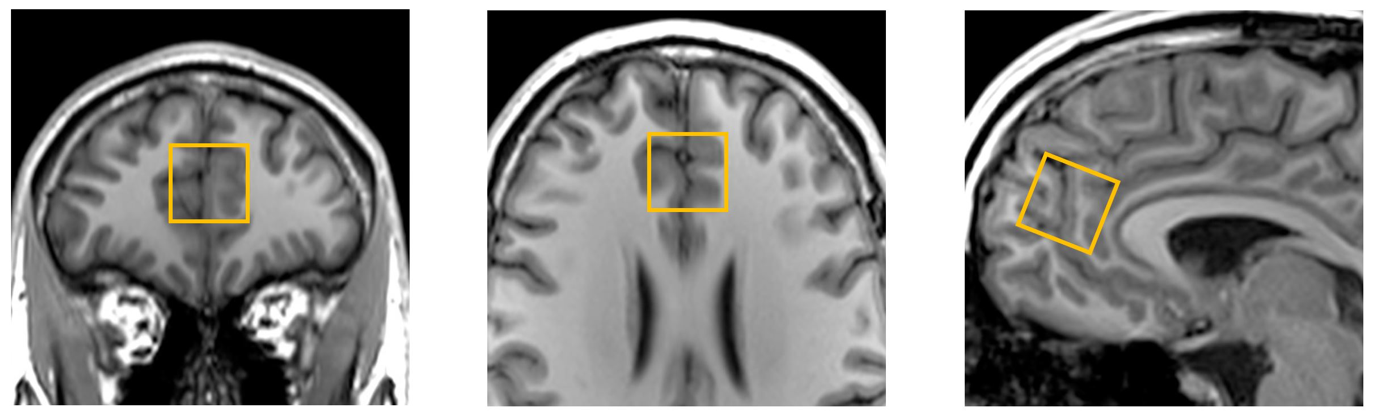

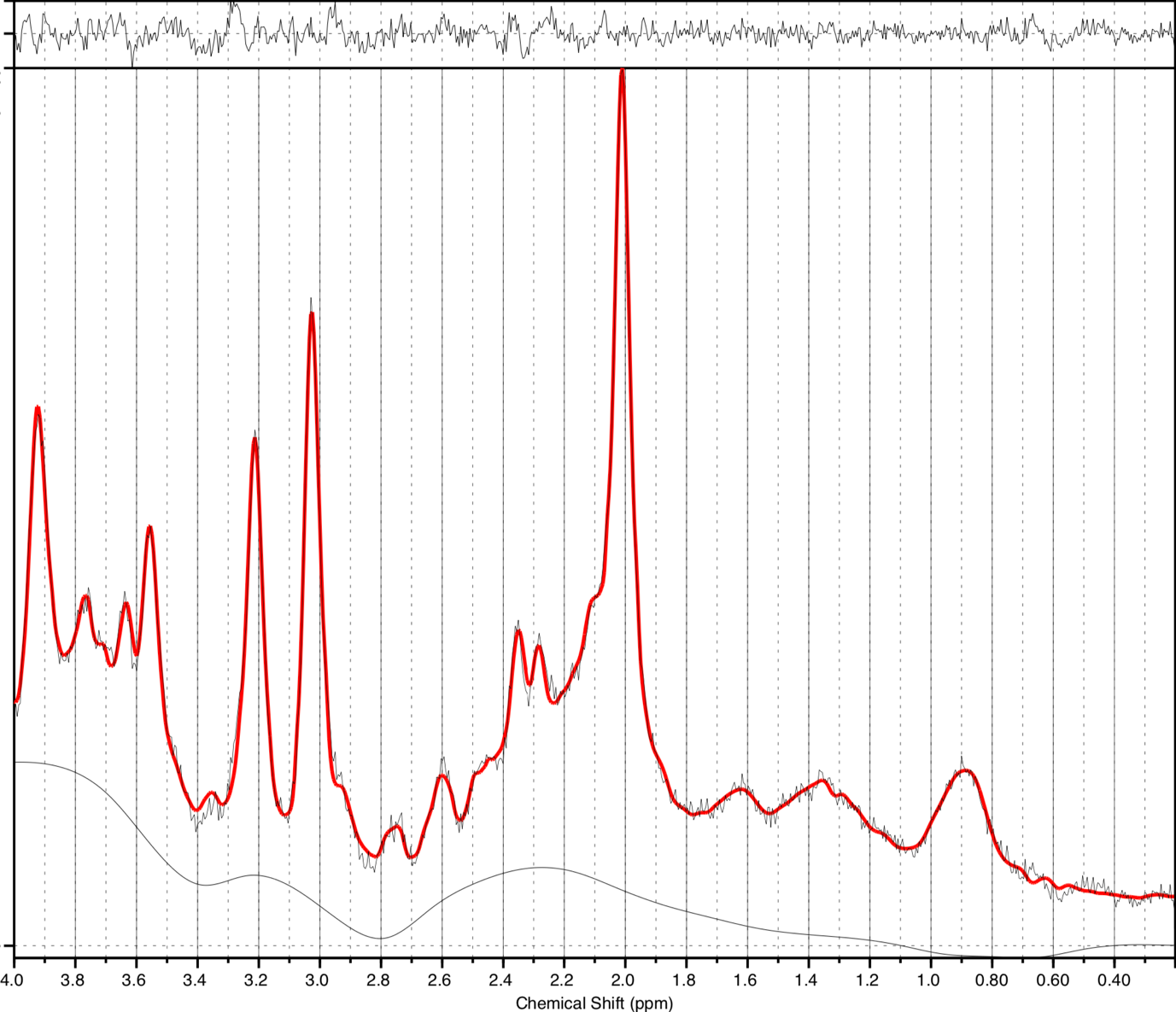


Supplementary Figure 1 – ^1^H-MRS voxel position and example spectrum in the anterior cingulate cortex


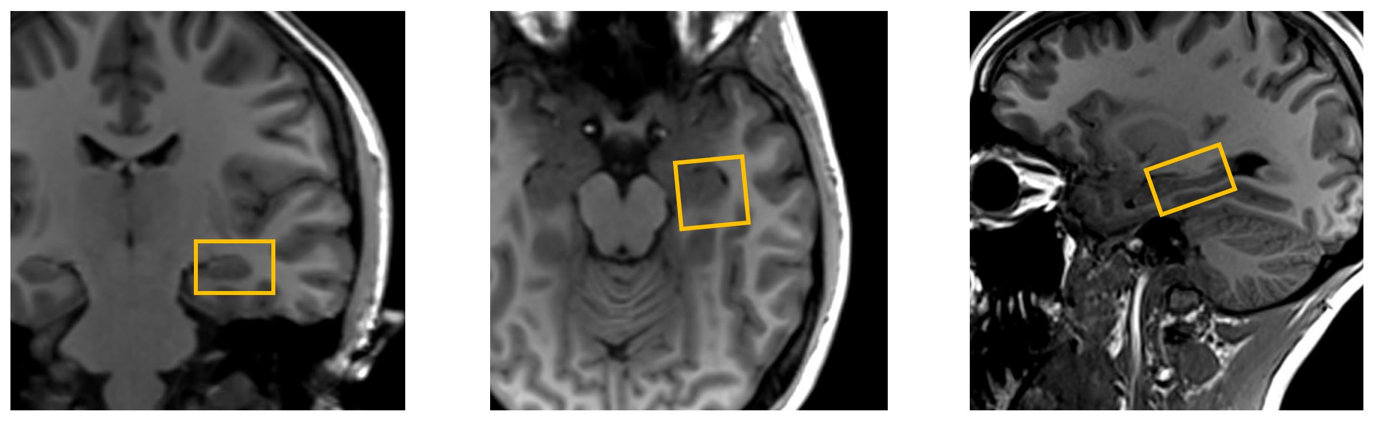

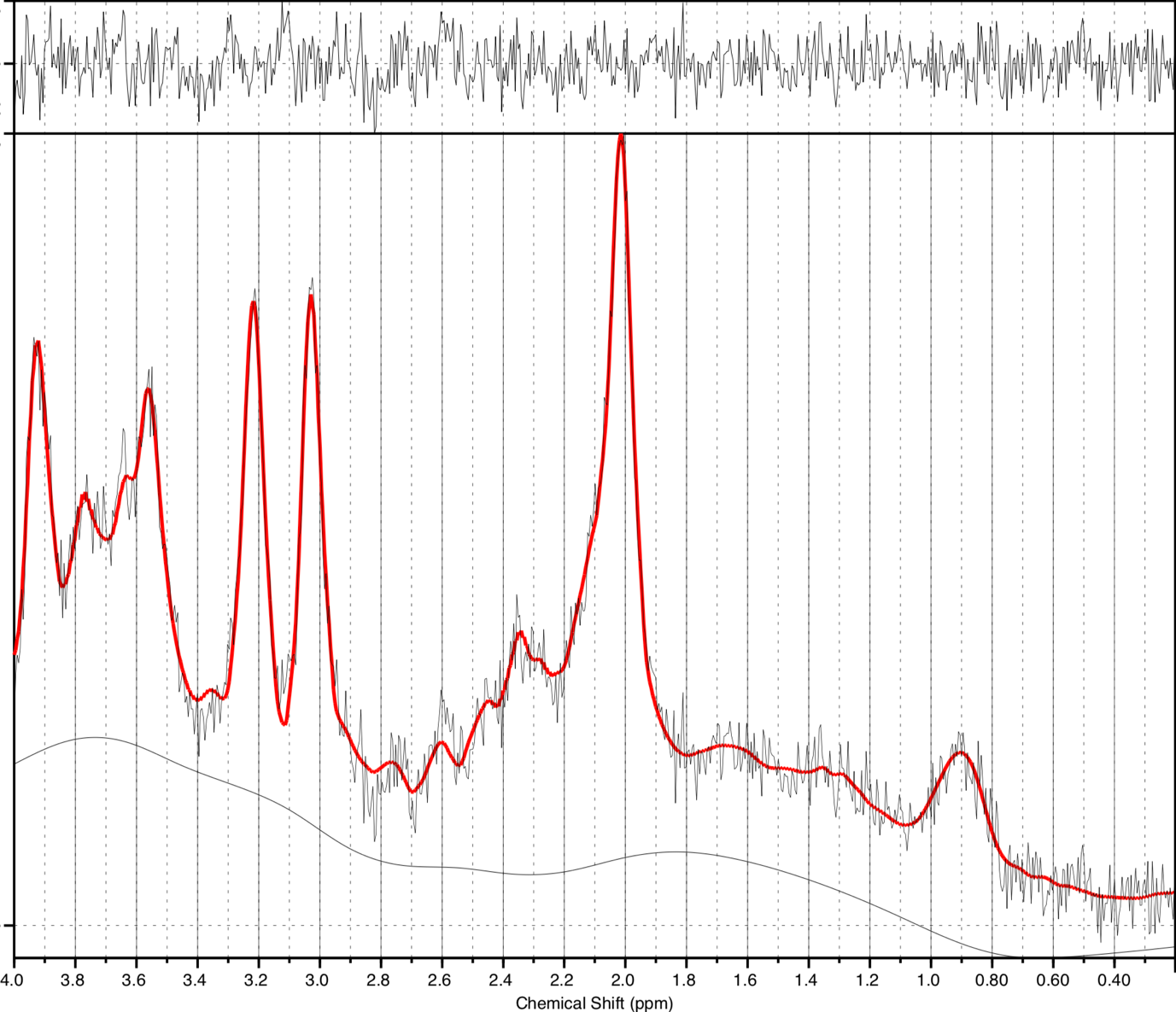


Supplementary Figure 2 – ^1^H-MRS voxel position and example spectrum in the left hippocampus

Supplementary Figure 3 **– Significant positive relationship between [^11^C]UCB-J DVR and Glx/Cr levels in the ACC in healthy volunteer group (HV, n = 22, r = 0.72, p = 0.0002).**

Supplementary Figure 4 **– No significant relationship between [^11^C]UCB-J DVR and Glx/Cr levels in the ACC in schizophrenia group (SCZ, n = 18, r = 0.05, p = 0.86).**

Supplementary Figure 5 **– No significant relationship between [^11^C]UCB-J DVR and NAA/Cr levels in the ACC in healthy volunteer group (HV, n = 22, r = 0.07, p = 0.77, Bonferroni-corrected alpha-threshold = 0.0125).**

Supplementary Figure 6 **– No significant relationship between [^11^C]UCB-J DVR and NAA/Cr levels in the ACC in schizophrenia group (SCZ, n = 18, r = 0.47, p = 0.048, Bonferroni-corrected alpha-threshold = 0.0125).**

Supplementary Figure 7 **– Significant positive relationship between [^11^C]UCB-J DVR and Glx/Cr levels in the left hippocampus in healthy volunteer group (HV, n = 22, r = 0.48, p = 0.03).**

Supplementary Figure 8 **– No significant relationship between [^11^C]UCB-J DVR and Glx/Cr levels in the left hippocampus in schizophrenia group (SCZ, n = 17, r = 0.16, p = 0.53).**

Supplementary Figure 9 **– No significant relationship between [^11^C]UCB-J DVR and NAA/Cr levels in the left hippocampus in healthy volunteer group (HV, n = 22, r = 0.23, p = 0.31).**

Supplementary Figure 10 **– No significant relationship between [^11^C]UCB-J DVR and NAA/Cr levels in the left hippocampus in schizophrenia group (HV, n = 18, r = -0.17, p = 0.50).**

Supplementary Figure 11 **– No significant relationship between [^11^C]UCB-J V_T_ and Glu/Cr levels in the anterior cingulate cortex (ACC) in healthy volunteer group (HV, n = 22, r = 0.33, p = 0.14).**

Supplementary Figure 12 **– No significant relationship between [^11^C]UCB-J V_T_ and Glu/Cr levels in the anterior cingulate cortex (ACC) in schizophrenia group (SCZ, n = 18, r = 0.24, p = 0.34).**

Supplementary Figure 13 **– No significant relationship between [^11^C]UCB-J V_T_ and Glx/Cr levels in the anterior cingulate cortex (ACC) in healthy volunteer group (HV, n = 22, r = 0.39, p = 0.07).**

Supplementary Figure 14 **– No significant relationship between [^11^C]UCB-J V_T_ and Glx/Cr levels in the anterior cingulate cortex (ACC) in schizophrenia group (SCZ, n = 18, r = 0.007, p = 0.98).**

Supplementary Figure 15 **– No significant relationship between [^11^C]UCB-J V_T_ and NAA/Cr levels in healthy volunteer group (HV, n = 22, r = -0.18, p = 0.43).**

Supplementary Figure 16 **– No significant relationship between [^11^C]UCB-J V_T_ and NAA/Cr levels in schizophrenia group (SCZ, n = 18, r = -0.22, p = 0.37).**

Suuplementary Figure 17 **– Significant positive relationship between [^11^C]UCB-J V_T_ and Glu/Cr levels in the hippocampus in healthy volunteer group (HV, n = 22, r = 0.47, p = 0.03).**

Supplementary Figure 18 **– No significant relationship between [^11^C]UCB-J V_T_ and Glu/Cr levels in the left hippocampus in schizophrenia group (SCZ, n = 17, r = 0.13, p = 0.61).**

Supplementary Figure 19 **– No significant relationship between [^11^C]UCB-J V_T_ and Glx/Cr levels in the left hippocampus in healthy volunteer group (HV, n = 22, r = 0.22, p = 0.32).**

Supplementary Figure 20 **– No significant relationship between [^11^C]UCB-J V_T_ and Glx/Cr levels in the hippocampus in schizophrenia group (n = 17, r = -0.11, p = 0.68).**

Supplementary Figure 21 **– No significant relationship between [^11^C]UCB-J V_T_ and NAA/Cr levels in the left hippocampus in healthy volunteer group (HV, n = 22, r = -0.14, p = 0.53).**

Supplementary Figure 22 **– No significant relationship between [^11^C]UCB-J V_T_ and NAA/Cr levels in the left hippocampus in schizophrenia group (SCZ, n = 18, r = 0.02, p = 0.93).**
